# Improved-Throughput Traction Microscopy Based on Fluorescence Micropattern for Manual Microscopy

**DOI:** 10.1371/journal.pone.0070122

**Published:** 2013-08-01

**Authors:** Kai Liu, Yuan Yuan, Jianyong Huang, Qiong Wei, Mingshu Pang, Chunyang Xiong, Jing Fang

**Affiliations:** 1 Academy for Advanced Interdisciplinary Studies, Peking University, Beijing, China; 2 Department of Mechanics and Aerospace Engineering, College of Engineering, Peking University, Beijing, China; University of California, San Diego, United States of America

## Abstract

Traction force microscopy (TFM) is a quantitative technique for measuring cellular traction force, which is important in understanding cellular mechanotransduction processes. Traditional TFM has a significant limitation in that it has a low measurement throughput, commonly one per TFM dish, due to a lack of cell position information. To obtain enough cellular traction force data, an onerous workload is required including numerous TFM dish preparations and heavy cell-seeding activities, creating further difficulty in achieving identical experimental conditions among batches. In this paper, we present an improved-throughput TFM method using the well-developed microcontact printing technique and chemical modifications of linking microbeads to the gel surface to address these limitations. Chemically linking the microbeads to the gel surface has no significant influence on cell proliferation, morphology, cytoskeleton, and adhesion. Multiple pairs of force loaded and null force fluorescence images can be easily acquired by means of manual microscope with the aid of a fluorescence micropattern made by microcontact printing. Furthermore, keeping the micropattern separate from cells by using gels effectively eliminates the potential negative effect of the micropattern on the cells. This novel design greatly improves the analysis throughput of traditional TFM from one to at least twenty cells per petri dish without losing unique advantages, including a high spatial resolution of traction measurements. This newly developed method will boost the investigation of cell-matrix mechanical interactions.

## Introduction

Cellular traction force is the physical force exerted by cells on the extracellular matrix. It is implicated in a variety of vital cellular physiological and pathological processes including contraction [1], [2], migration [3]–[6], wound healing [7]–[9], metastasis [10] and angiogenesis [11]. The quantitative evaluation of traction forces is becoming essential for better understanding the cellular mechanotransduction mechanism and precisely describing various cellular functions. For example, a significant difference in traction forces exists between metastatic and non-metastatic cells, which can be used as a biophysical marker to characterize cellular metastatic potential [12].

At present, traction force microscopy (TFM) is an efficient and reliable method to determine the cellular traction forces acting on a flat flexible substrate [13]–[15]. The key section of traditional TFM is the acquisition of a pair of “force loaded” and “null force” microscopy images (hereafter referred to as FL image and NF image, respectively) [16]. An FL image is a fluorescence image of the deformation of soft substrates caused by cells, and an NF image is a fluorescence image of undeformed elastic substrates taken at the same location by detaching the cell through trypsinization or similar methods [17]. Although TFM has been successfully conducted in various cell mechanical experiments, it has an obvious drawback in that the measurement throughput is usually low because there are no valid methods for acquiring multiple NF images [16], [18]. In the traditional TFM protocol, though plenty of isolated cells exist on the surface of relatively large substrates in one dish, and given that fluorescence microbeads are randomly dispersed, it is nearly impossible to return to the exact position of each cell from the last view field when capturing its NF image. Thus, only the traction force of the last cell can be obtained in a single TFM dish.

The low traction measurement throughput limitation of traditional TFM may be noticeable in statistical analysis. It takes quite a bit of work to prepare the large number of TFM dishes needed to collect enough sample data. Different cell culture conditions among dishes induce difficulties in analyzing experimental results. Due to its disadvantages of low cell utilization rate, time/labor consumption and uneconomic expenses, mass-manufacture of traditional TFM dishes may be replaced by developing improved-throughput traction measurement solutions. Some recent reports discuss solutions using motorized microscope [19] and surface micropattern technique [20]. However, these two methods also have limitations such as the requirement of expensive instruments or the low spatial resolution of traction forces. Therefore, a more convenient, simpler and high-spatial-resolution improved-throughput TFM method is urgently needed.

In this study, we present an improved-throughput TFM method using the microcontact printing technique and surface chemical modifications to construct a new measurement device based on the traditional TFM dish. In the device, the fluorescence micropattern existing on a glass cover slip underneath a flat polyacrylamide (PAA) gel substrate serves as a coordinate system for recording the position of each seeded cell, and beads are chemically linked to the surface of PAA gels for tracking cell-mediated displacements. The micropattern is easily fabricated by standard microcontact printing. The beads on the gel surface have no significant influence on cell morphology, cytoskeleton, adhesion, and proliferation. This modified traction measurement device enables us to manually capture multiple pairs of NF and FL images with a common inverted fluorescence microscope without motorized stages. An average of twenty or more traction force fields of HeLa cells in a single petri dish is successfully reconstructed in our experiments, greatly improving the measurement throughput of traditional TFM while maintaining its unique advantages such as the high spatial resolution of traction forces and convenient fabrication. This improved-throughput TFM will vastly boost cellular traction force researches, in particular has the potential to promote the traction force study of rare cells from clinical samples.

## Results and Discussion

### Improved-throughput TFM Device

**Figure 1:**
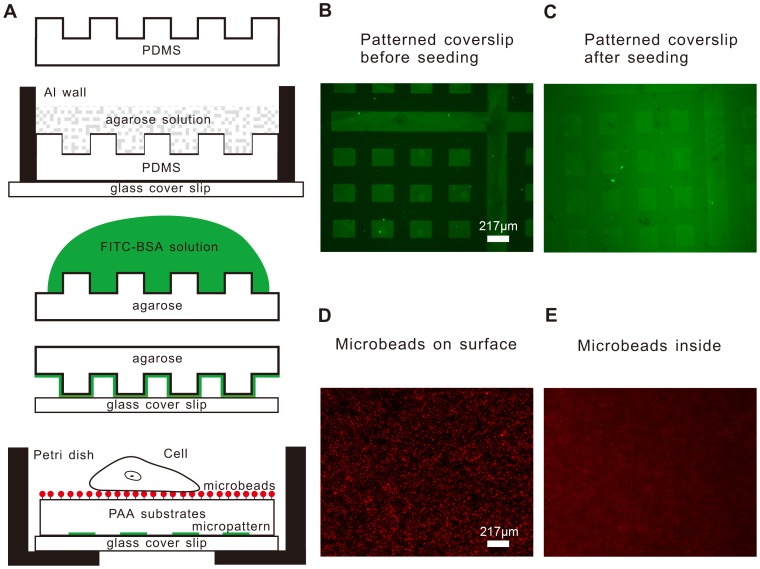
Schematic diagram showing the fabrication of the improved-throughput TFM device. **A)** The procedure of microcontact printing on the coverslip and the structure of the new designed device. **B)** Fluorescence image of the micropattern on the cover slip before seeding cells. **C)** The micropattern observed through the culture medium after seeding cells. **D)** The microbeads on the surface of the PAA gels observed by the fluorescence microscope. **E)** The image of microbeads inside gels.

We designed a novel device that can be used to measure multiple traction forces in one petri dish. Fig. 1A illustrates the procedure for fabricating this improved-throughput device. The PDMS piece replicated the featured silicon wafer as the mold for the agarose stamp. The FITC-BSA was perfectly absorbed onto the surface of the agarose stamp as the print ink. The glass cover slip patterned by the agarose stamp was used as the bottom of a cover slip sandwich where the PAA gel was able to polymerize. This micropattern is composed of regularly dispersed 217×172 µm^2^ rectangles spaced 200 µm apart (Fig. 1B). The micropattern has a strong fluorescent intensity, rendering it distinguishable for observers to read the relative position information of each small rectangle through the substrates (Fig. 1C). Fig. 1A shows the final fabricated device where the green micropattern is on the surface of the bottom coverslip and the red microbeads are bound to the top of substrate with amido bonds catalyzed by EDC and Sulfo-NHS reagents. The structure of the presented device is quite different from that of traditional TFM devices with embedded microbeads in the substrates and a blank coverslip underlying the substrates. Fig. 1D shows the fluorescence image of surface microbeads, and Fig. 1E shows fluorescence image of microbeads embedded in the PAA gels. It is clear that the background noise in Fig. 1D is much weaker than that in Fig. 1E, thus the signal to noise ratio is stronger to make the second focusing much easier in the case of surface microbeads.

In this procedure, an agarose stamp was chosen to complete the print work due to its convenient fabrication and ability to print numerous times [22]. Because two different fluorescent substances, the red microbeads and the green FITC-BSA micropattern, were physically separated by the approximately 70-µm-thick PAA gels (Fig. 1A), the cross-color phenomenon did not occur when changing the exciting light to capture different fluorescence images (Fig. 1C, Fig. 1D). In traditional TFM, the microbeads are first mixed into the PAA gels and then the sandwich is reversed to sink microbeads to the apical surface of the substrates. As shown in Fig. 1E, the background optical noise from microbeads in the lower focus plane is so large enough that it may affect the accuracy of second focusing to the previous plane. The method of chemically linking the microbeads to the surface of the PAA gels ensured there were no microbeads within the PAA gels, thus avoiding background fluorescence noise in other focus planes. This method guaranteed that the FL and NF images were captured in the same focus plane, preventing illusional displacement in DIC calculation.

### No Distinguished Influence of the Beads on the Gel Surface on the HeLa Cells

In our method, chemically linking the beads to the PAA gel surface replaced the traditional practice of mixing the beads into the gel. We implemented several experiments to verify whether beads on the surface have influence on the HeLa cells.

**Figure 2:**
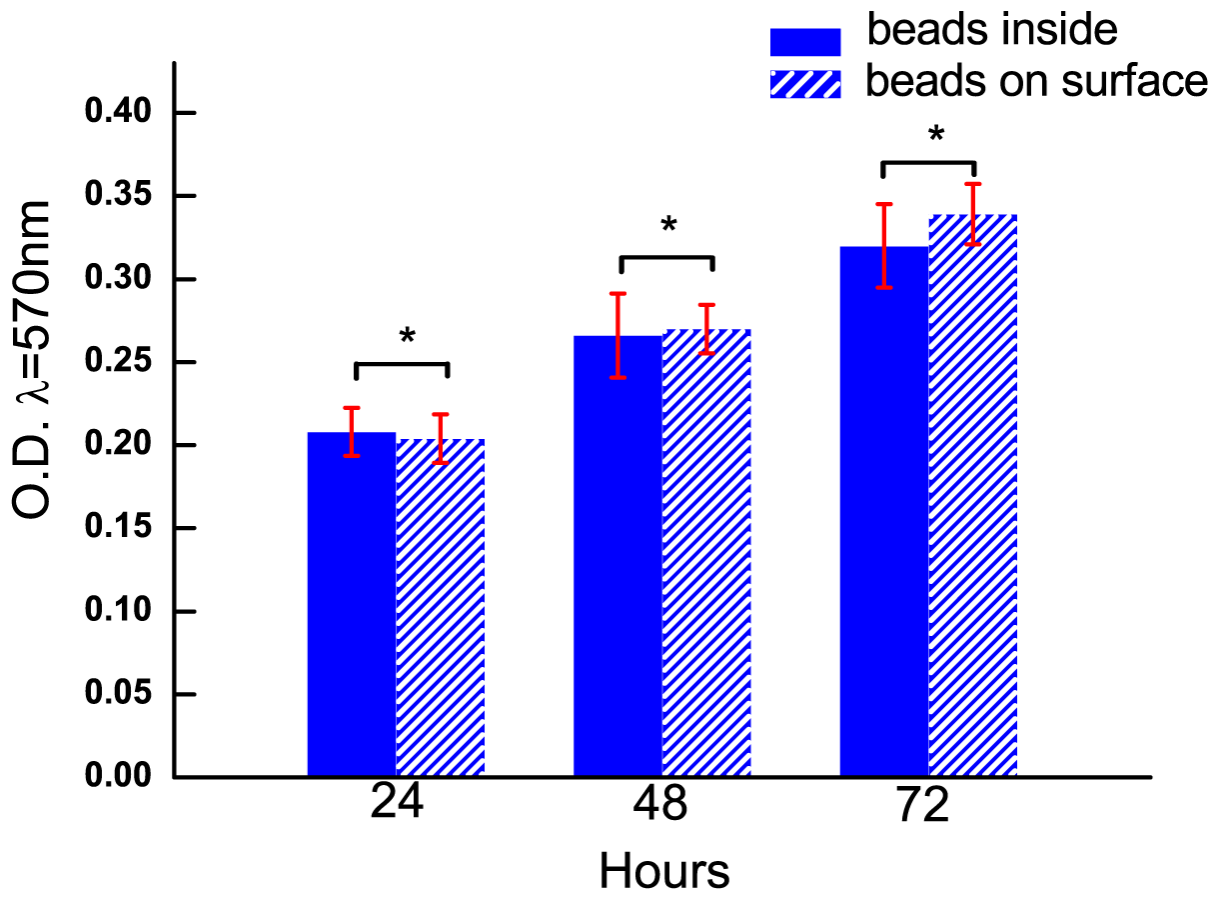
Results of MTT assay on substrate with beads inside and on surface. The optical density at wavelength of 570 nm of each sample at 24 h, 48 h, and 72 h was detected. Bars represent mean ± standard deviation. Two-tailed t-test was performed for statistical comparisons (n = 3, *represents *p*>>0.05).

**Figure 3:**
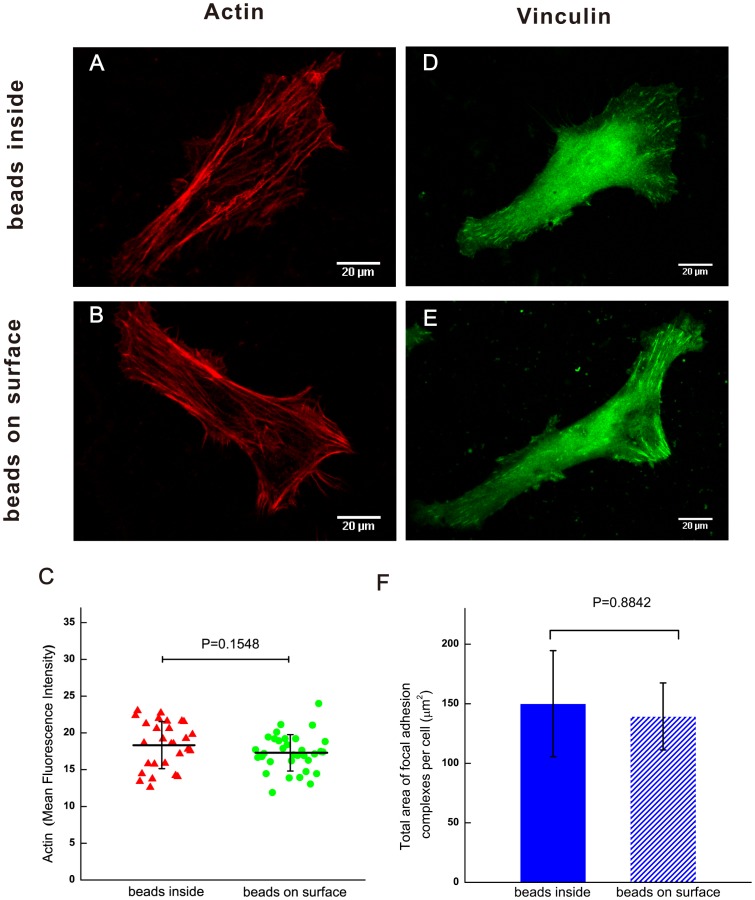
Immunostaining of cells on substrate of different topography. **A)** Representative immunofluorescence confocal microscopic images of the F-actin (red) of cells on substrate with beads inside. **B)** Representative immunofluorescence confocal microscopic images of the F-actin (red) of cells on substrate with beads on surface. **C)** Statistical quantification of the mean fluorescence intensity of actin within the HeLa cells on substrate with different positioned beads (n = 28 for beads inside, n = 34 for beads on surface). **D)** Representative immunofluorescence confocal microscopic images of the vinculin (green) with beads inside. **E)** Representative immunofluorescence confocal microscopic images of the vinculin (green) with beads on surface. **F)** Comparison of total vinculin area on PAA gels with beads inside and beads on the gel surface (n = 22 for the former, n = 20 for the latter). Bars represent mean ± standard deviation. Two-tailed t-test was performed for statistical analysis in both **C)** and **F)**.

The effect of the beads on cell growth was investigated by MTT assay as shown in the Fig. 2. Both groups are obviously seen to promote the cell growth at a similar trend with no significant difference (*p*>>0.05). Fig. 3 shows the immunostaining results of actin cytoskeleton and vinculin, an important protein of focal adhesion complexes. Most of HeLa cells on both substrates appear like a fusiform and align numerous actin stress fibers along the long axis of the cell (Fig. 3A, Fig. 3B). It is obvious that the little vinculin strips were mainly formed in the lamellipodia of cells in Fig. 3D and Fig. 3E. The cell on the substrate with beads on surface has a 3637±895 µm^2^ area and 0.414±0.123 shape factor, while the cell on the substrate with beads inside has a 3535±1122 µm^2^ area and 0.394±0.117 shape factor. Using the two-tailed t-test statistical analysis, we found there no significant difference in the cell area (n = 100 in each set, *p* = 0.4452), shape factor (n = 100 in each set, *p* = 0.2033), F-actin expression (*p* = 0.1548, Fig. 3C) and vinculin expression (*p* = 0.8842, Fig. 3F). We also performed other cell like Sprague-Dawley Rat MSC, NIH 3T3 fibroblast and Sprague-Dawley Rat cardiomyocyte on the substrate with beads on surface versus beads inside. The cell area, shape factor and cytoskeleton were unaffected by the topography of substrate with beads on the gel surface (Figure S1, Table S1).

These data above suggest that the beads on surface have no significant influence on cells compared to the control with traditional beads inside. It needs to mentioned that the surface of substrate with beads on top or beads inside is uniformly covered with collagen type I presenting a high density of ligand. The 260∼410 nm width of collagen fibrils is near to the size of beads [36]. The beads are covered with the collagen fibrils so that it may decrease the nanocues of the beads to some extent. There are abundant adhesion ligands existing on the collagen [37] [38], which can promote cell adhesion and spread. These two strong effects of collagen may overcome the topographic stimuli of beads on surface.

### Beads as a Tracker of the Deformation of the PAA Gels

**Figure 4:**
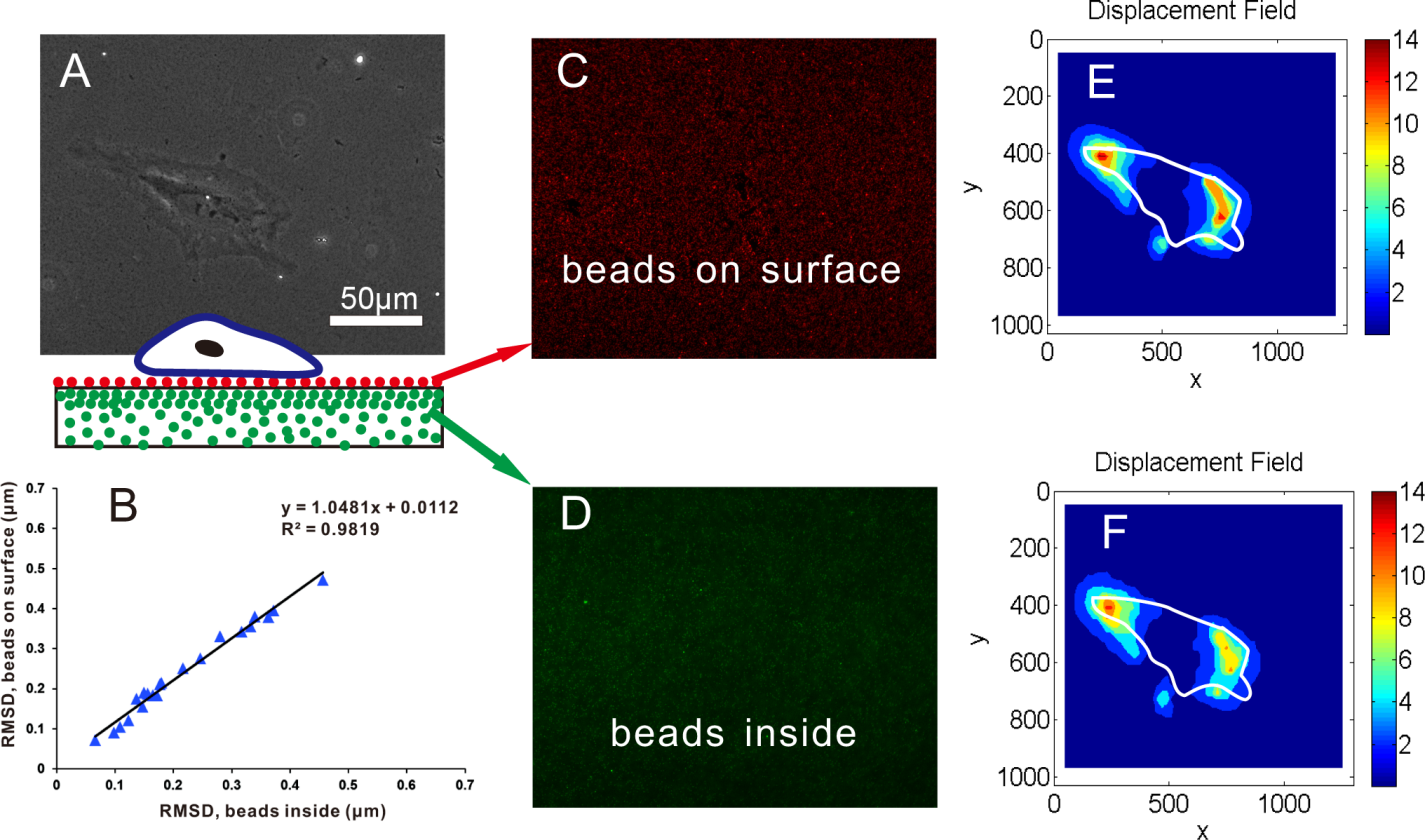
Experimental validation of beads on surface as an indicator of substrate deformation. **A)** Phase-contrast image of HeLa cell on the PAA gels (beads were both mixed inside and linked on surface of the gels, as illustrated in the inset). **B)** Scatter plot of RMSD computed for 22 cells utilizing fluorescence images of the beads on surface (vertical axis) and beads inside (horizontal axis). **C)** Fluorescence image of beads on surface. **D)** Fluorescence image of beads inside gels. **E)** Displacement field was calculated using the fluorescence image of beads on surface before and after cell removal by NaOH solution. **F)** Displacement field was calculated using the fluorescence image of beads inside before and after cell removal by NaOH solution. The solid white line stood for the cell outline in the both **E)** and **F)**.

In the traditional TFM, the topmost beads embedded in the PAA gels can track the deformation of substrates caused by the seeded cell [24]. We developed an approach to link 0.2 µm beads to PAA surface in improved-throughput TFM. To ensure that the new positioned beads can also indicate the substrate deformation like the traditional methods, we seeded the HeLa cells on substrate prepared with red fluorescent beads on surface and green fluorescent beads inside (Fig. 4A, inset). We captured the red fluorescence images of beads linked to the gel surface (Fig. 4C) and kept the same focus plane to capture the green fluorescence images of beads inside gels (Fig. 4D) before and after cell detachment by NaOH solution. We computed the displacement fields (Fig. 4E and Fig. 4F) from the corresponding images of beads utilizing DIC algorithm. In this experiment, the root mean square displacement (RMSD) values were calculated using the two sets of beads, inside and on surface of the substrate, for each cell (Fig. 4B) by MATLAB. The results displayed excellent agreement between displacement fields indicated by the two sets of beads, with a total correlation coefficient (R^2^) of 0.9819 of 22 cells with RMSD from 0.1 µm to 0.47 µm (Fig. 4B). On basis of these data, we conclude that the deformation recorded from beads on gel surface is equal to the deformation of the gel. The beads on the gel surface can be an effective indicator of the substrate deformation.

### Multiple Pairs of NF and FL Fluorescence Images

**Figure 5:**
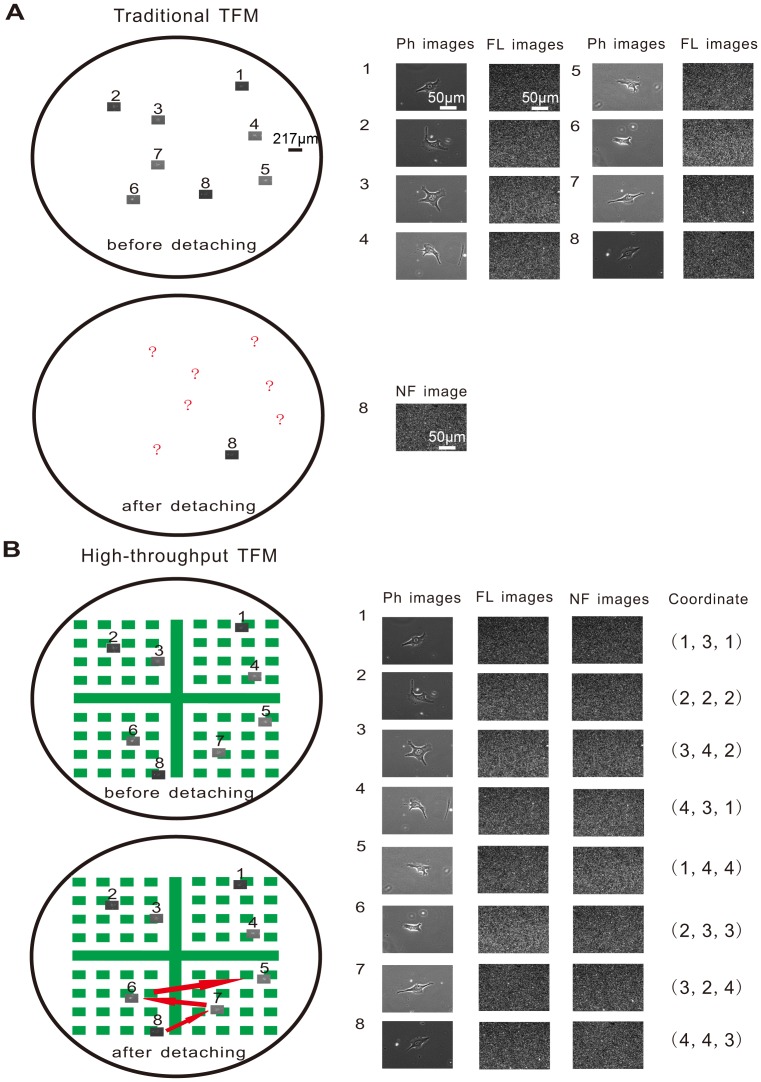
Multiple pairs of NF and FL fluorescence images. **A)** Many FL images had been captured before cell detachment while the NF image of only the last cell was captured after detaching all cells, as other cells could not be found in the traditional TFM. **B)** Utilizing coordinate system, multiple pairs of NF and FL images were captured in sequence by going back to the original position in the improved-throughput TFM. The circle stands for the PAA substrate. The small rectangle in the circle represents the view field using the 40× objective.

Fig. 5 shows the results of image acquisition from two different TFMs. Fig. 5A is a schematic diagram indicating the reason of low efficiency while NF images are acquired using traditional TFM [24]. There were eight FL images captured before detaching the seeded cells on the PAA gels. Because there was no way to record where the eight cells were, not all of the corresponding NF images could be captured after the cell removal, i.e. only the last cell’s NF image could be recorded since its view field still stayed. In Fig. 5B, the micropattern, a fluorescence rectangle array with a large inset cross dividing the array into four quadrants similar to a coordinate system, was placed under the PAA gels substrate. When the cells were within the range of the micropattern, taking the cell numbered one for instance, a specific coordinate was assigned to it (1, 3, 1), indicating the cell was directly above the rectangle in the first row of the third column in the first quadrant. During FL image capture, each cellular position on the micropattern was recorded as shown in Fig. 5B. After all cells were detached by using NaOH solution, multiple NF fluorescence images could be captured using the recorded position information. Eight pairs of NF and FL fluorescence images are shown in Fig. 5B, representative of the total pairs captured from a single petri dish.

The protocol above involves in basic functions of common fluorescence microscope, which is also generalizable to other microscope devices. We implemented the same experiments on other microscope systems with different CCD, strictly following our protocol. These systems are Olympus IX81 with ANDOR iXon 885 EMCCD, Zeiss Axio Observer A1 with TUCSEN TCC-1.4HICE-11 CCD and Nikon Ti-E with HAMAMATSU C9100-13 EMCCD. Using these microscopes, we also easily gained multiple pairs of NF and FL fluorescence images.

In conclusion, the positioning function of the current micropattern is very usable and efficient to facilitate the achievement of the improved-throughput of traction measurements.

### Elimination of the Stage Shift

**Figure 6:**
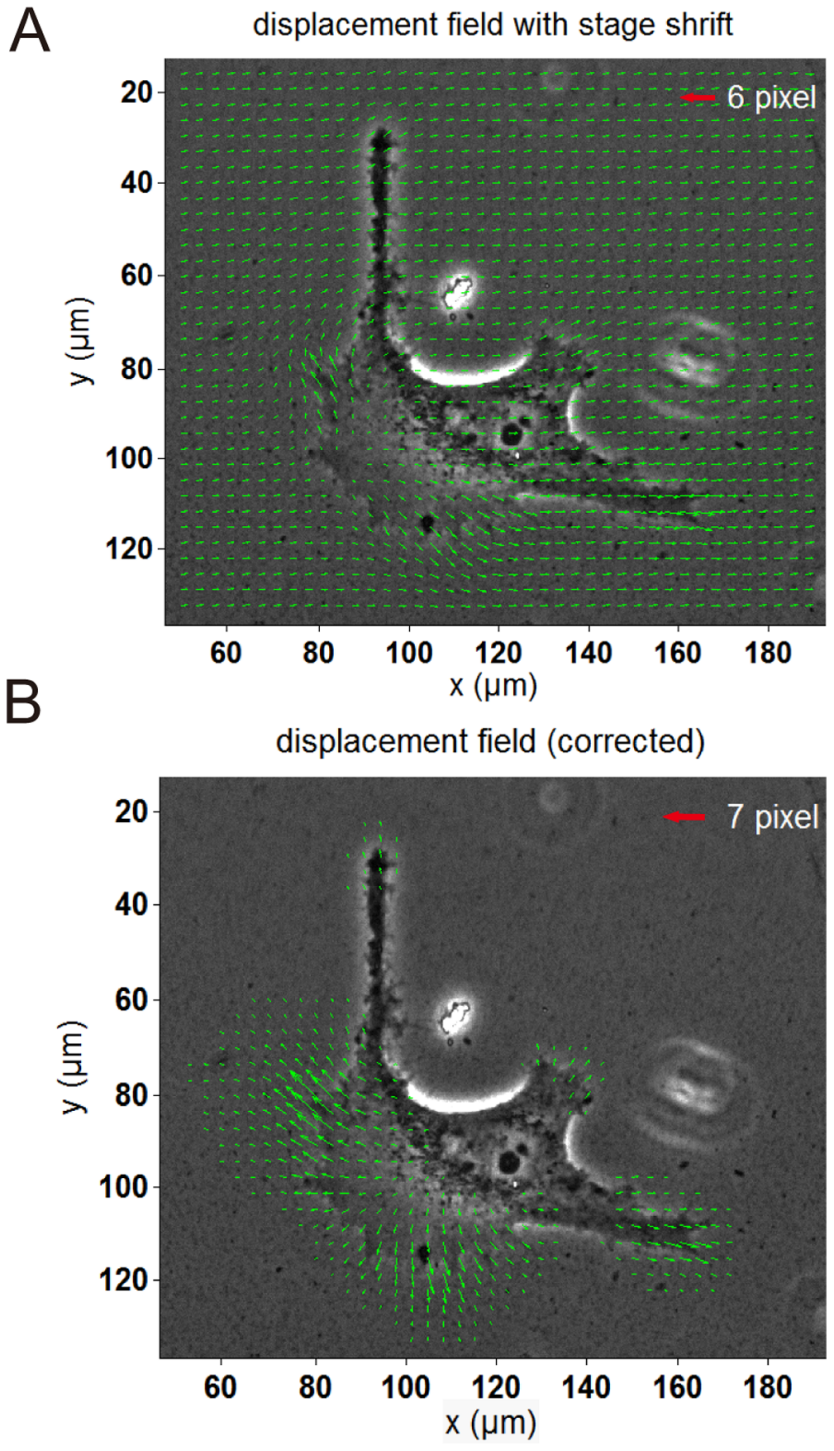
Eliminating the stage shift in the improved-throughput TFM. **A)** Apparent stage shift in the original displacement field. **B)** The actual displacements caused by HeLa cells after correction by the image processing algorithm.

During the NF image acquisition process, manual adjustment was required to match the view field of the current image to that of the corresponding FL image; exact replication of the original position was not possible. An average rigid displacement of 0.3673±0.1694 µm, arising from the stage shift, was observed in each pair of images processed by DIC algorithm. The uniform rigid displacement in the displacement fields is shown in Fig. 6A. The traction forces calculation is a typical ill-posed inverse problem, which is subjected to the noise in the displacement fields [34]. And the Boussinesq solution only works for deformation fields with zero-average. Therefore, we utilized a simple and improved image processing algorithm to eliminate average rigid displacement induced by the stage shift among images before applying an integral Boussinesq solution to recover the cellular traction forces. The substrate deformation generated by cells decreased quickly, thus the displacement of the marginal area, far away from the cell, should be theoretically zero, while it was actually not zero because the tiny stage shift induced a uniform displacement throughout the whole field. For this reason, the mean displacement of the marginal area was equivalent to the stage shrift displacement. Meanwhile, the stage shrift also superimposed this displacement upon the region underneath the cell. Therefore, it was reasonable to use algorithm to subtract the average displacement of the marginal area, standing for the shrift displacement, from the previous whole displacement field to eliminate the side-effect of stage shrift and obtain the true displacement field of substrate deformation caused by the cell. Fig. 6B shows a displacement field of the real substrate deformation generated by the cellular traction force after image processing. The maximum displacement, 7 pixels, which was previously lost in the rigid movement of the substrate, has become larger than that in Fig. 6A. After we revised the displacement in each pair of images, the RMSD value decreased from 0.3348 µm to 0.1375 µm. Comparing the displacement field in Fig. 6A and that in Fig. 6B, the latter one has no displacement on the marginal area. These data imply that the uniform rigid displacement induced by the stage shrift apparently disappears from the cellular displacement field.

To estimate the accuracy of the obtained displacement field using the improved-throughput TFM, we also examined the distribution of correlation coefficient values of all sample points in the investigated cell area. In DIC theory, the correlation coefficient is commonly used as an indicator of the quality of displacement conversion [34]. We found that the correlation coefficient of most sample points was greater than 0.9, and the cumulative distribution of these sample points took up 92% of the total sample points. It’s the same level compared with those found if the matched images are acquired without manual adjustment of the view field, as is the usual practice in traditional TFM. These results suggest that the calculated displacement field in our novel design excellently represents the actual movement of the microbeads generated by cellular traction forces. Additionally, the slight stage shift has no significant negative influence on the accuracy of results.

### Improved-throughput Traction Force Fields

**Figure 7:**
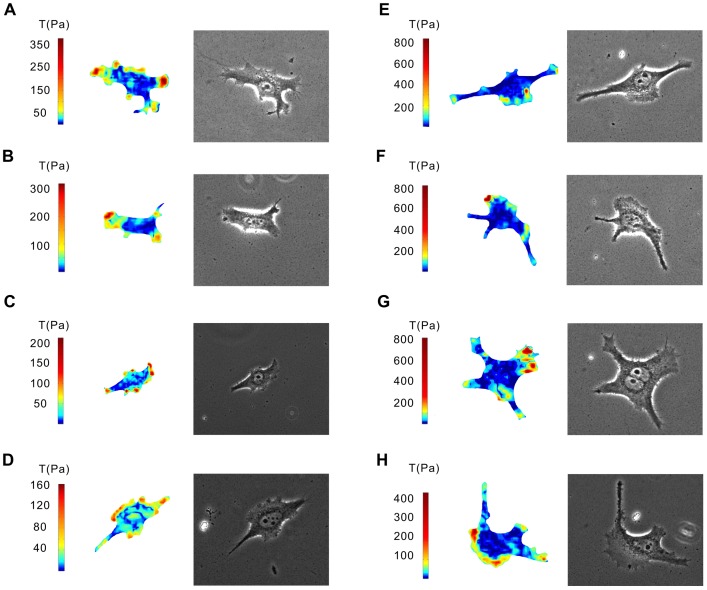
The results of improved-throughput measurements. Each panel was composed of a colorimetric bar, traction force field and phase-contrast image. The recovered traction fields of **A**, **B**, **C**, **D**, **E**, **F**, **G** and **H** were only a part of the total traction force fields in one petri dish.

Fig. 7 presents some of the traction force fields of HeLa cells in one dish recovered using a MATLAB program. Generally speaking, at least twenty fields could be reconstructed in a single TFM dish, obviously superior to one or a few that can be acquired using the traditional TFM assay [16], [19]. According to the measurement conditions, the number of total traction fields in one dish can be as many as several hundred in theory.

The low throughput of traditional TFM has been a concern in most cell mechanics laboratories. Recently, several published papers have aimed to address this problem, including a motorized microscope method by using automated microscopic mapping [19] and an indirect microcontact printing method for printing fluorescence-conjugated fibronectin micropattern to the surface of PAA gels [20]. However, the former method required expensive microscope equipment and special software to accomplish automated microscopic imaging. Our method utilized a common inverted microscope without any need for the facility and software above to reduce the experimental expenses and simply realized the goal of capturing the multiple fluorescence images. In the microcontact printing method, fluorescent fibronectin dots covalently connected to the surface of substrate composed a grid micropattern in which cells were constrained to adhere. The displacement field was determined from the deformation between the traction-loaded micropattern and an assumed traction-free micropattern. Some studies have shown that cells cultured on different adhesive micropatterns show different arrangements of cytoskeleton and focal adhesions; cellular traction force distributions will change in response to these [18], [39]. It brought a potential challenge of this method how eliminating the side-effect of micropattern on cellular cytoskeleton when measuring traction forces. In addition, the spatial resolution of this method was limited; the distance between fluorescent dots was designed to be larger than 5 µm because closer than that, the displacement of each dot would be disturbed by the traction force exerted on neighboring ones. The limitation was still unsolved by its current computational approach. In our work, the fluorescence micropattern existed under the elastic substrate rather than on its surface so that cells did not contact the micropattern, enabling us to effectively eliminate the above-mentioned problems and make the experimental results clear. The spatial resolution of the recovered traction force in our technique was consistent with that of traditional TFM, which achieved a sub-pixel (1 pixel equaling to 0.1675 µm) level of resolution so that more detailed information could be obtained to explain the cell activity [32], [40], [41].

In conclusion, we have improved traditional TFM by using a fluorescence micropattern array and surface-bound microbeads to realize the improved-throughput traction measurements. The significant achievement of our method is that at least twenty traction force fields can be acquired in a single dish within one hour as compared to the one or several fields using traditional TFM. Thus, our method greatly reduces the workload needed to obtain vast traction force measurements and increases the utilization rate of cells. Additionally, the confusion in traction forces measurements resulting from the unavoidable differences of experiment conditions among batches is now removed because all data of new method come from one dish. These strong points are essential to biological statistical analysis. On top of these advantages, our convenient, low expensed and effective method is hopeful to be an alternative solution in the laboratories, which are being trapped in the low throughput measurements. With the superiority of simple, convenience, high-speed and cost-efficiency, our improved-throughput TFM may accelerate biophysical research of stem cell differentiation and cancer invasion, especially for effective mechanical characterization of cells from clinical samples [42]–[44]. Future work will be focused on determining the biomechanical markers of diagnostics of various cancers using this improved-throughput TFM.

## Materials and Methods

### Microcontact Printing

The fluorescence micropatterned glass cover slip, the key section of our improved-throughput TFM, was fabricated by standard microcontact printing [21]. The featured silicon wafer, fabricated by photolithography, was placed in a vacuum chamber and exposed overnight at room temperature to 1H, 1H, 2H, 2H-perfluorocydecyltriethoxysilane vapor (Sigma). Polydimethylsiloxane (PDMS, Sylgard 184, Dow Corning), mixed in a 1∶10 ratio of curing agent to base, was used to replicate the characteristics of the silicon wafer. The PDMS piece (cutting beforehand) was enclosed using aluminum foils and the agarose solution (8% (w/v) agarose (Invitrogen) in deionized water) was poured into the chamber, then the chamber was heated to 121°C in an autoclave (MLS-3750, SANYO). After the solution cooled naturally, the agarose stamp was formed. The high concentration of agarose afforded stable, replicated features from the PDMS pieces and maintained the integrity of the top features during the microcontact printing process [22]. Simultaneously, the 22×22 mm^2^ glass cover slip was treated with a 4% (v/v) solution of 3-aminopropyltrimethoxysilane (Sigma) in acetone (Beijing Chemical Works) for 30 minutes, 0.5% (v/v) glutaraldehyde (Beijing Yili Company) in phosphate buffered saline (PBS) (Gibco) for 30 minutes and washed with deionized water for 10 minutes. The cover slips were covalently bonded to the PAA gels with the help of glutaraldehyde. The agarose stamp adsorbed a solution of fluorescein isothiocyanate-labeled bovine serum albumin (FITC-BSA, Invitrogen) at a concentration of 100 µg/mL for 30 minutes. The agarose stamp was gently placed on the activated cover slip, avoiding any movement for approximately 5 minutes, and then carefully peeled off from the cover slip.

### PAA Substrate Preparation and Surface Conjugation of Microbeads

The PAA gel was prepared per the published protocol [1]. In this experiment, each 100 µL reaction solution contained 25 µL of 20% (w/v) acrylamide (Invitrogen), 5 µL of 2% (w/v) bis-acrylamide (Invitrogen), 0.5 µL of 10% (w/v) aminopropylsilane (AP, Sigma), 1 µL of 5% (v/v) tetramethylethylenediamine (TEMED) (Fluka) and 66.5 µL of deionized water. The thickness of the PAA gel was approximately 70 µm, and the final concentration of acrylamide and bis-acrylamide were 5% and 0.1%, respectively. The Young’s modulus of the PAA substrates used in this study was approximately 2.5 kPa [23], and the Poisson’s ratio was set as 0.5 according to previous studies [24], [25]. After polymerization, the PAA gel surface was successively covered by a 0.2 µm rhodamine carboxylate-modified microbeads aqueous solution (diluted at 1∶400) for 15 minutes, 1-ethyl-3-[3-dimethylaminopropyl] carbodiimide, hydrochloride (EDC)/Hydroxy-2,5-dioxopyrrolidine- 3-sulfonicacid (Sulfo-NHS) solution (3.8 and 7.6 mg/mL, Invitrogen and Sigma, respectively) [26] prepared in 2-(N-Morpholino)ethanesulfonic acid (MES) pH 5.5 (Sigma) for 2 hours and PBS pH 7.4 for 2 hours to finally achieve chemical conjugation of the microbeads with gels surface. In some experiments, the 0.2 µm FITC-conjugated microbeads were also added to the PAA gels in volume ratio 1∶100.

### Cell Culture

To successfully seed HeLa cells, the PAA gel surface was derivatized with sulfosuccinimidyl-6-[4′-azido-2′nitrophenylamino] hexanoate (sulfo-SANPAH) (Amresco) under ultraviolet radiation, washed by 50 mM HEPES (Invitrogen) pH 8.5 and covalently linked with 0.2 mg/mL collagen type I(Gibco) in PBS by incubation overnight at 4°C.

Cells were cultured in Dulbecco’s modified Eagle’s medium (DMEM, Invitrogen) with 10% (v/v) fetal bovine serum (Biochrom), 1‰ (v/v) penicillin/streptomycin (Amresco) and 1 mM sodium pyruvate solution (Sigma). The PAA gels were sterilized with UV radiation for 30 minutes and incubated with 2 mL of culture medium for 30 minutes prior to use. HeLa cells were seeded onto the PAA gels at an appropriate density and cultured in an incubator with 5% CO_2_ and 95% humidity at 37°C.

### Cell Proliferation Assay

HeLa cell proliferation was determined by a 3-(4,5-dimethylthiazol-2-yl)-2,5-diphenyl-tetrazolium bromide (MTT) assay [27]. HeLa cells were seeded onto the 22×22 mm^2^ substrate gels covered with the microbeads at a density of 3000 cells per dish. After cell adhered on the substrate for 2 hours at 5% CO_2_ and 37°C, the dish was washed by 2 mL culture medium to ensure no cell adhered on the regions outside the gel. Next, the cells were incubated at 5% CO_2_ and 37°C for 24, 48 and 72 h, respectively. A standard MTT assay was performed by the end of each experiment interval. The supernatants were discarded and cells were washed by PBS. The formazan crystals were dissolved in dimethylsulfoxide (Sigma) after 3 hours of treatment with 5 mg/mL of MTT (Sigma). Optical density (OD) at a wavelength of 570 nm was measured using a Model 680 microplate reader (Bio-Rad). For each time point, three dishes with the microbeads inside containing similar cells number were regarded as controls.

### Immunofluorescence Staining for Vinculin and Actin

HeLa cells seeded on the corresponding substrates (beads on surface versus beads inside) for 24 hours were immunostained after fixation with 4% paraformaldehyde (Sigma) for 30 minutes, permeabilization with 0.1% Triton X-100 (Amresco) for 5 minutes, blocking with 1% Bovine Serum Albumin (Sigma) for 60 minutes. Samples were washed with PBS before every step. The cells were incubated for 30 minutes with rhodamine-phalloidin following manufacturer’s instruction (Invitrogen) for actin immunostaining. The cells were incubated with a mouse anti-vinculin monoclonal primary antibody (1∶100 dilution) (Abcam) overnight at 4°C and with a goat anti-mouse IgG FITC-conjugated secondary antibody (1∶100 dilution) (Abcam) for 60 minutes at room temperature.

### Immunostaining Image Acquisition and Statistical Analysis

Stained cells were imaged using the 40× (1.2 NA) water objective on the Zeiss 710 confocal laser scanning microscope, and images were collected using Zen 2010 software.

Both the cell spread area (A) and the perimeter (P) were computed using Image J (National Institutes of Health) to calculate actin immunostaining images. Shape factor, an indicator of how branched the cell is, is defined as the ratio 4πA/P^2^, varying from 1 for a circle shape to 0 for a highly branched shape [28]–[30]. A minimum of 100 cells in control and experiment sets was randomly chosen to calculate their shape factor.

The mean fluorescence intensity of actin stained cell images was analyzed by Image-Pro plus (Media Cybernetics). The vinculin region was separated from the original vinculin stained images through image calculator function in the Image J. And the total area of vinculin region per cell was determined by Image-Pro plus. To standardize the fluorescence intensity measurements, the time of image capturing, image exposure time, image intensity gain, image offset, and image black level were optimally adjusted at the beginning and kept constant for all experiments [31].

All data were compared using the two-tailed Student’s t-test and reported as the mean ± standard deviation using Microsoft Excel (Microsoft). Difference in means was considered significant at *p*<0.05.

### Improved-throughput Traction Force Measurements

Experimental images were acquired using an inverted microscope with a 40× (0.60 NA) objective (Olympus IX71) and DVC-1312 camera (DVC company). After the HeLa cells adequately spread and applied traction force on the underlying substrates, we began to capture various expected images. We chose the cell in the center of the view field and captured its phase-contrast image (Ph image) to investigate the boundary of cellular traction forces. The image of red fluorescence microbeads on the surface of the PAA gel substrate under this cell was also recorded by changing the bright light to 580 nm exciting light. The focus plane was turned down and the exciting wavelength was decreased to 497 nm so as to capture the green FITC-BSA micropattern image and determine the corresponding position of the cell in the micropattern. We acquired multiple FL fluorescence images following the above protocol before detaching seeded cells. Once cells were removed by NaOH solution, we captured the NF fluorescence images of the previous cells by means of the images of the green micropattern and the previously obtained position information.

A digital image correlation algorithm (DIC) was applied to process pairs of NF and FL fluorescence images [32], [33]. The in-plane displacement of the sampling point at 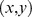 is determined by mathematically matching the grey intensity between the reference subset centered at 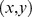 in the FL image and the corresponding target subset in the NF image. A so-called cross-correlation coefficient of grey intensity between subsets has been widely used in DIC as an indicator of subset matching and is defined as.
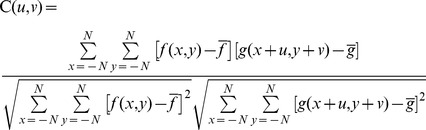
(1)where 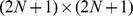
is the size of the subset, 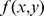 and 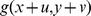 are the grey intensities at 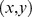 in the FL image and at 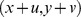 in the reference NF image, respectively; 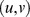
are the offset components along *x* and *y* between the two subsets, respectively; and 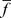
and 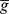 are the average grey intensity values of the pixels in the subsets A series of correlation coefficients, 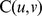, are calculated when matching the reference subset in the FL image with the potential target subsets in the NF image by switching 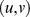 pixel by pixel. In equation (1), the maximum correlation coefficient represents the most potentially accurate position of the reference subset in the NF image. Hence, one can easily find the corresponding location of the reference subset centered at 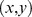 in the NF image by positioning at the maximum of these correlation coefficients, where the corresponding offset indicates the displacement of the subset centered at 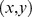 in *x* and *y*, respectively. Due to the stage shift existing in the experiments, a correcting algorithm was applied to subtract the tiny shift so that the traction force field could be recovered by applying an integral Boussinesq solution to the corrected displacement field [34], [35].

All the data were presented as the mean ± standard deviation computed by Microsoft Excel (Microsoft).

## Supporting Information

Figure S1 **Immunostaining of Sprague-Dawley Rat MSC on substrate with different topography. A)** Representative immunofluorescence confocal microscopic images of the F-actin (red) of MSCs on substrate with beads inside. **B)** Representative immunofluorescence confocal microscopic images of the F-actin (red) of MSCs on substrate with beads on surface. **C)** Statistical quantification of the mean fluorescence intensity of actin within the MSCs on substrate with different positioned beads (n = 19 for each sets). **D)** Representative immunofluorescence confocal microscopic images of the vinculin (green) with beads inside. **E)** Representative immunofluorescence confocal microscopic images of the vinculin (green) with beads on surface. **F)** Comparison of total vinculin area on PAA gels with beads inside (n = 14) and beads on the gel surface (n = 16). Bars represent mean ± standard deviation. Two-tailed t-test was performed for statistical analysis in both **C)** and **F)**.
(TIF)

Table S1 **The appearance comparison of other cells on two substrates.** The other three kinds of cells were cultured on the substrate with beads on surface or beads inside. The difference of the each set in area and shape factor was investigated using the two-tailed t-test.
(DOC)
