# Supplementary figures and images for: Improved-Throughput Traction Microscopy Based on Fluorescence Micropattern for Manual Microscopy
Source: PLoS One. 2013 Aug 1;8(8):e70122. doi: 10.1371/journal.pone.0070122 (PMC3731345; doi:10.1371/journal.pone.0070122)

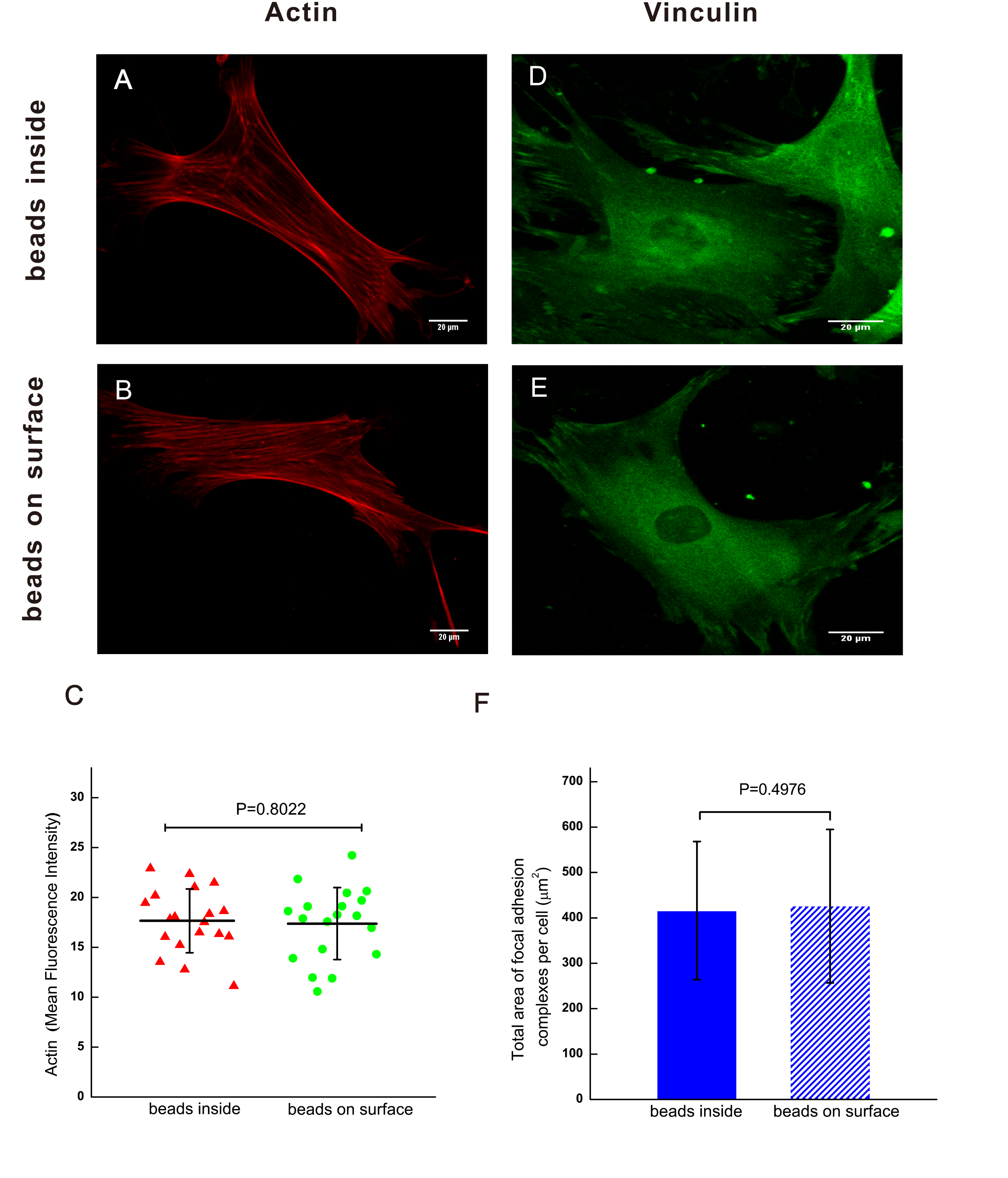

Supplement: Figure S1 — Immunostaining of Sprague-Dawley Rat MSC on substrate with different topography. A) Representative immunofluorescence confocal microscopic images of the F-actin (red) of MSCs on substrate with beads inside. B) Representative immunofluorescence confocal microscopic images of the F-actin (red) of MSCs on substrate with beads on surface. C) Statistical quantification of the mean fluorescence intensity of actin within the MSCs on substrate with different positioned beads (n = 19 for each sets). D) Representative immunofluorescence confocal microscopic images of the vinculin (green) with beads inside. E) Representative immunofluorescence confocal microscopic images of the vinculin (green) with beads on surface. F) Comparison of total vinculin area on PAA gels with beads inside (n = 14) and beads on the gel surface (n = 16). Bars represent mean ± standard deviation. Two-tailed t-test was performed for statistical analysis in both C) and F). (TIF) [file pone.0070122.s001.tif]
