# Supplementary material for: Improved-Throughput Traction Microscopy Based on Fluorescence Micropattern for Manual Microscopy
Source: PLoS One. 2013 Aug 1;8(8):e70122. doi: 10.1371/journal.pone.0070122 (PMC3731345; doi:10.1371/journal.pone.0070122)
Supplement: Table S1 — The appearance comparison of other cells on two substrates. The other three kinds of cells were cultured on the substrate with beads on surface or beads inside. The difference of the each set in area and shape factor was investigated using the two-tailed t-test. (DOC) [file pone.0070122.s002.doc]

**Table S1. The appearance comparison of other cells on two substrates.**

| cell | Sprague-Dawley Rat MSC | | NIH 3T3 | | Sprague-Dawley Rat cardiomyocyte | |
| --- | --- | --- | --- | --- | --- | --- |
| substrate | beads on surface | beads inside | beads on surface | beads inside | beads on surface | beads inside |
| area(μm^2^) | 2685±360 | 2849±652 | 2273±507 | 2355±600 | 1949±720 | 1984±845 |
| *p* value | 0.2502 (n=19) | | 0.7094 (n=13) | | 0.8765 (n=10) | |
| significant difference | no | | no | | no | |
| shape factor | 0.260±0.086 | 0.253±0.082 | 0.36±0.09 | 0.36±0.08 | 0.33±0.08 | 0.32±0.13 |
| *p* value | 0.7948 (n=19) | | 0.2044 (n=13) | | 0.8726 (n=10) | |
| significant difference | no | | no | | no | |

The other three kinds of cells were cultured on the substrate with beads on surface or beads inside. The difference of the each set in area and shape factor was investigated using the two-tailed t-test
